# Synergistic biofilm formation by the coexistence of nontypeable *Haemophilus influenzae* and *Moraxella catarrhalis* reduces amoxicillin efficacy

**DOI:** 10.1186/s12866-026-05420-x

**Published:** 2026-07-16

**Authors:** Nafisa Khamis Umar, Ryo Ueda, Tatsuya Shiga, Taku Fujishiro, Yoshimi Onishi, Tomoko Kamiyama, Hideki Sakatani, Kazuya Mizobata, Shunji Tamagawa, Muneki Hotomi, Masamitsu Kono

**Affiliations:** 1 Department of Otorhinolaryngology-Head and Neck Surgery, Wakayama Medical University, 811-1 Kimiidera, Wakayama, 641- 8510 Japan; 2 FUJIFILM Toyama Chemical Co., Ltd., 14-1, Kyobashi 2-Chome, Chuo- Ku, Tokyo, 104-0031 Japan

**Keywords:** *Haemophilus influenzae*, *Moraxella catarrhalis*, Biofilm, Amoxicillin, Respiratory quinolones

## Abstract

**Background:**

Nontypeable *Haemophilus influenzae* (NTHi) and *Moraxella catarrhalis* are recognized as the key pathogens that cause respiratory tract infections, and both are capable of forming biofilms. Although interspecies biofilm formation has been described, the impact of co-culture conditions on antimicrobial responsiveness remains incompletely defined. In this study, we examined biofilm formation by co-culturing NTHi and *M. catarrhalis* and evaluated their antimicrobial responsiveness to amoxicillin and alternative clinically relevant antibiotics, including sequential exposure following initial amoxicillin treatment, in an in vitro model.

**Results:**

Biofilm formation was quantitatively evaluated using the crystal violet staining method with a 96-well pin replicator. Twenty clinical isolates of *M. catarrhalis* were first cultured for 24 h to assess their biofilm-forming capacities, which varied considerably among strains, with some producing robust biofilms. To further examine interspecies interactions, *M. catarrhalis* and NTHi were co-cultured at different CFU ratios, revealing that increasing the proportion of *M. catarrhalis* in the inoculum enhanced overall biofilm production compared with either species alone. Additionally, exposure to selected antibiotics showed that respiratory quinolones were associated with greater reductions in biofilm biomass and viable bacteria within co-culture-derived biofilms compared with amoxicillin, both during direct treatment and following prior amoxicillin exposure, under the experimental conditions tested.

**Conclusion:**

Co-culture of NTHi and *M. catarrhalis* was shown to significantly enhance biofilm formation compared with either culture species alone. In particular, a higher proportion of *M. catarrhalis* was associated with increased biofilm production. These findings suggest that polymicrobial coexistence may contribute to reduced antimicrobial responsiveness through enhanced biofilm formation.

**Supplementary Information:**

The online version contains supplementary material available at https://doi.org/10.1186/s12866-026-05420-x.

## Background

Nontypeable *Haemophilus influenzae* (NTHi) and *Moraxella catarrhalis* are major pathogens responsible for acute otitis media (AOM) and acute rhinosinusitis. Increasing antimicrobial resistance has been documented in both NTHi and *M. catarrhalis*, including rising rates of ampicillin resistance reported in surveillance studies and global epidemiological analyses [1, 2]. In NTHi, resistance to β-lactam antibiotics is mediated by both β-lactamase production, primarily TEM-1 and ROB-1 enzymes, and structural alterations in penicillin-binding protein 3, leading to the emergence of β-lactamase-negative ampicillin-resistant (BLNAR) strains. The prevalence of these resistance mechanisms varies considerably across geographic regions [3, 4]. In contrast, the majority of *M. catarrhalis* isolates produce BRO type β-lactamases (BRO-1 and BRO-2), which efficiently hydrolyze aminopenicillins and confer high-level resistance to ampicillin, while having limited activity against extended-spectrum cephalosporins [5–7]. NTHi has emerged as a predominant causative pathogen in cases of intractable AOM.

Bacterial biofilms are structured communities of microorganisms embedded in a self-produced extracellular matrix composed of polysaccharides, proteins, lipids, and extracellular DNA [8]. Within this matrix, bacteria exhibit altered physiology and reduced susceptibility to antimicrobial agents, partly due to limited antibiotic penetration, metabolic heterogeneity, and the presence of persister cells [9]. In NTHi, extracellular DNA has been identified as a key structural component of biofilms [10]. Recent studies have demonstrated that mature NTHi biofilms contain Z-form extracellular DNA stabilized by DNABII family proteins, which enhances biofilm stability and resistance to enzymatic degradation [11]. These properties are thought to contribute to the persistence of NTHi on mucosal surfaces and its association with recurrent or chronic infections [12, 13]. These mechanisms have been specifically demonstrated in NTHi biofilms, where reduced metabolic activity and structural protection contribute to antimicrobial tolerance [10, 12].

We previously reported that NTHi increases biofilm formation in response to sub-minimum inhibitory concentration (sub-MIC) levels of amoxicillin [14]. Although β-lactamase production is recognized as an important mechanism of reduced susceptibility, biofilm-associated tolerance mechanisms, such as restricted drug diffusion and survival of non-replicating cells, remain incompletely understood.

In clinical settings, NTHi frequently coexists in the nasopharynx with other otopathogens. Clinical persistence and relapse after antibiotic treatment are commonly observed in polymicrobial infections [15–19]. Experimental studies have demonstrated that NTHi forms structurally integrated polymicrobial biofilms with respiratory pathogens, including *M. catarrhalis*, in which close spatial interactions contribute to enhanced stability and resistance phenotypes [20]. Biofilm formation has therefore been proposed as a contributing factor to treatment failure; however, the mechanistic roles of polymicrobial biofilms in antibiotic failure remains incompletely defined. *M. catarrhalis* expresses several virulence factors implicated in biofilm development, including UspA adhesins, type IV pili, and outer membrane vesicles (OMVs), and has been shown to promote NTHi survival within mixed-species biofilms colonizing the human nasopharynx [21, 22]. Colonization with *M. catarrhalis* occurs early in life and is more frequently associated with polymicrobial rather than monomicrobial AOM [23, 24].

Despite this clinical relevance, most experimental studies have evaluated antibiotic effects using single-species NTHi biofilms, and the impact of co-existence of *M. catarrhalis* on amoxicillin efficacy has not been fully elucidated. Here, we evaluate the synergistic effect of biofilm formation resulting from the co-existence of NTHi and *M. catarrhalis*, and we investigate the reduction in amoxicillin efficacy under these polymicrobial conditions.

## Methods

### Bacterial strains and growth condition

As in our earlier work [14, 25, 26], we used IH-202 in this study, a β-lactamase non-producing ampicillin susceptible strain of NTHi. We also used 20 clinical isolates of *M. catarrhalis* obtained from pediatric patients with AOM. The strains used in this study were handled in three defined generations. First-generation consisted of isolates originally obtained from patients and stocked by freezing at -80 °C. Second-generation was laboratory-stocked strains derived from the first-generation. Third-generation was prepared by culturing a single colony of the second-generation stocks in liquid medium followed by freezing aliquots for experimental use. All experiments were performed using these third-generation stocks to ensure uniform inoculum preparation and to minimize variability associated with differences in recovery after thawing.

### Antimicrobial susceptibility test and antibiotic concentrations

The antimicrobial activity of amoxicillin, cefditoren, garenoxacin, and tosufloxacin was assessed following the microbroth dilution protocol outlined by the Clinical and Laboratory Standards Institute (M07-A11, 2018). Antibiotic powders of garenoxacin and tosufloxacin were kindly provided by FUJIFILM Toyama Chemical Co., Ltd. Briefly, bacterial suspensions were adjusted to approximately 5 × 10^5^ colony forming units (CFU)/mL in supplemented brain heart infusion medium (sBHI; Difco Laboratories, Detroit, USA) and inoculated into 96-well microtiter plates containing serial twofold dilutions of each antibiotic. Plates were incubated at 37 °C under 5% CO_2_ for 18–20 h. The minimum inhibitory concentration (MIC) was defined as the lowest antibiotic concentration that completely inhibited visible growth [14].

### Biofilm quantification using a 96-pin replicator system

Biofilm development by NTHi and *M. catarrhalis* was quantified according to the assay previously established in our laboratory, with minor modifications [14, 25]. Briefly, bacterial suspensions were adjusted to an optical density at 600 nm (OD₆₀₀) of 0.5 in sBHI medium containing 15 µg/mL hemin and 15 µg/mL NAD (Sigma, St. Louis, USA). The same sBHI medium was used for both monoculture and co-culture biofilm assays to maintain standardized experimental conditions and allow direct comparison among assays. Cultures were shaken at 180 rpm at 37 °C, diluted tenfold in fresh sBHI, and dispensed (200 µL per well) into U-bottom 96-well plates (Nunc 262162, Thermo Fisher Scientific, Yokohama, Japan). Each plate was covered with a removable 96-pin lid (Watson Co., Tokyo, Japan) and incubated with gentle agitation (80 rpm) for 24–72 h at 37 °C, with lids transferred daily to fresh medium when prolonged incubation was required. Based on our previous studies [14, 25], shaking conditions were adopted to ensure reproducible biofilm formation.

After incubation, the pins were rinsed three times with sterile phosphate-buffered saline to eliminate planktonic cells and then air-dried. Adherent biofilms were stained with 0.1% crystal violet for 15 min at room temperature [25, 27–29]. Excess dye was removed by washing, and the bound stain was solubilized in 30% acetic acid. The optical density of the released dye at 570 nm was measured as an indicator of biofilm biomass, and this value (OD_570_) was defined as the Biofilm Index. Each condition was assayed in 8-fold replicates and repeated in at least two independent experiments.

### General experimental design

Biofilm formations of 20 clinical isolates of *M. catarrhalis* were evaluated using the 96-pin replicator assay, and we evaluated the synergistic biofilm formations of co-cultures of NTHi and *M. catarrhalis*. MC282 was selected for subsequent co-culture experiments because it demonstrated the most stable and reproducible biofilm production across repeated assays. This strain was confirmed to produce β-lactamase by a nitrocefin-based chromogenic cephalosporin assay (BML, Inc. Tokyo, Japan). After growing *M. catarrhalis* MC282 and NTHi IH202 separately to an OD₆₀₀ of 0.5 (corresponding to 1.2 × 10⁷ CFU/mL and 8.5 × 10⁸ CFU/mL, respectively), mixed bacterial suspensions were prepared at various ratios. Biofilm formation of the mixed bacterial suspensions was tested after 24 h and compared with that of monocultures. We evaluated the efficacy of antibiotics against biofilms formed by co-cultures of NTHi and *M. catarrhalis* using the 96-pin replicator assay.

### Antibiotic exposure

These procedures closely follow some used before and reported in the literature [11]. In summary, after the initial 24-hour (h) incubation, the 96-pin lids were relocated to fresh sBHI plates containing either antibiotics or control medium and incubated for another 24 h. This procedure was used to determine the ability of each antibiotic to prevent biofilm establishment and to remove preformed, mature biofilms. The assays were conducted using mixed-species biofilms containing approximately 2.16 × 10^5^ CFU of MC282 and 1.7 × 10^6^ CFU of IH202 (approximate CFU ratio = 1:8), which was selected as a representative high-biofilm-producing condition based on the trend observed in the current study. Antibiotic efficacy was examined at concentrations corresponding to 1/2 ×, 1 ×, and 2 × MICs, as well as clinically achievable levels. In addition to amoxicillin, which is the first-line drug for bacterial upper respiratory tract infections, cefditoren was selected as a representative oral β-lactam frequently used for pediatric AOM in Japan, while garenoxacin and tosufloxacin were chosen as respiratory quinolones with high tissue penetration into middle ear and sinus mucosa. Quinolones are reported to possess activity against NTHi biofilms and intracellular NTHi, supporting their evaluation in the present dual-species model [11]. The MIC values for NTHi applied in this study were based on our previous research and were as follows: amoxicillin MIC = 0.124 µg/mL; cefditoren (CDTR) MIC ≦ 0.0315 µg/mL; garenoxacin MIC ≦ 0.0315 µg/mL; and tosufloxacin MIC = 0.0625 µg/mL [14].

Antibiotic effects on biofilm formation and established biofilms were evaluated using four experimental conditions. These included assessment of (i) inhibition of biofilm formation, (ii) disruption of established biofilms, and (iii) the effect of sequential antibiotic exposure following initial amoxicillin treatment in co-culture. Detailed experimental conditions and timelines are described in the Results section.

The clinical concentrations used in the experiments were low concentration of amoxicillin in adult’s sinus (0.2 µg/ml), high concentration of amoxicillin in child’s middle ear fluid (5.8 µg/ml), garenoxacin at adult’s sinus concentration (6.01 µg/ml), tosufloxacin at child’s sinus concentration (0.96 µg/ml), and cefditoren at a child’s sinus concentration (0.684 µg/ml) [30–33].

### Viability of NTHi and *M. catarrhalis* in biofilm after antimicrobial treatments

For viable cell quantification, biofilm-associated bacteria were recovered by transferring the biofilm-coated pins into fresh sBHI medium and agitating at approximately 180 rpm for 1 h at 37 °C to facilitate detachment of adherent cells. This agitation condition was intentionally stronger than that used during biofilm formation (80 rpm) to enhance recovery of biofilm-associated bacteria. Although complete detachment cannot be guaranteed, the same recovery procedure was applied across all samples to allow comparative analysis. Co-cultures of IH202 and MC282 were prepared at a CFU ratio of *M. catarrhalis* : NTHi = 1:8 and grown in sBHI broth using 96-pin lids for 72 h: the first 24 h without antibiotics, the second 24 h with amoxicillin at 1 × MIC in fresh plates and medium, then the third 24 h with amoxicillin, garenoxacin, and tosufloxacin at 1 × MIC in a fresh plate and medium, at 37 °C with shaking at 80 rpm each. Following the 72 h incubation with sequential antibiotic exposure, viable IH202 and MC282 cells in the biofilms were harvested by immersing the pins in a fresh plate and incubating for 1 h at 37 °C with shaking. Collected samples were serially diluted 10-fold with sBHI in triplicates up to 12 series and cultured onto chocolate agar plates overnight at 37 °C under 5% CO_2_ environment. CFU were counted after overnight incubation under 5% CO_2_ at 37 °C for 18–40 h [34], allowing bacterial colony morphological distinction. Identification of the two species was performed based on colony morphology: *M. catarrhalis* formed opaque, white colonies, whereas NTHi produced greyish, translucent colonies. These morphological characteristics allowed reliable discrimination and separate CFU counting of each organism (representative images are provided in Fig. S1).

### Statistical analysis

All statistical analyses were performed using Prism 10.0 (GraphPad Software, Inc., La Jolla, CA). Comparisons between three or more groups were performed with Kruskal-Wallis tests and comparisons between two groups were made by Mann-Whitney U test. A *p* value of < 0.05 was considered to be statistically significant.

## Results

### Experimental design of antibiotic exposure conditions

To evaluate antibiotic effects under different conditions, we designed four experimental settings. In Exp. 1, the preventive effect of antibiotics on biofilm initiation was assessed by culturing bacteria for 24 h in the presence of each agent and comparing the results with antibiotic-free controls. In Exp. 2 and Exp. 3, the ability of antibiotics to disrupt established biofilms was examined. Biofilms were first allowed to mature for 24–48 h without antibiotics, followed by an additional 24-h exposure period with or without antimicrobial agents, corresponding to 24–48 h (Exp. 2) and 48–72 h (Exp. 3). In Exp. 4, we evaluated the effect of antibiotic treatment on amoxicillin-preconditioned biofilms in co-culture. Biofilms were formed for 24 h, exposed to amoxicillin for an additional 24 h, and then treated with alternative antibiotics. This sequential exposure design was intended to approximate a stepwise treatment scenario under experimental conditions.

### Biofilm production by *M. catarrhalis* clinical isolates

**Fig. 1:**
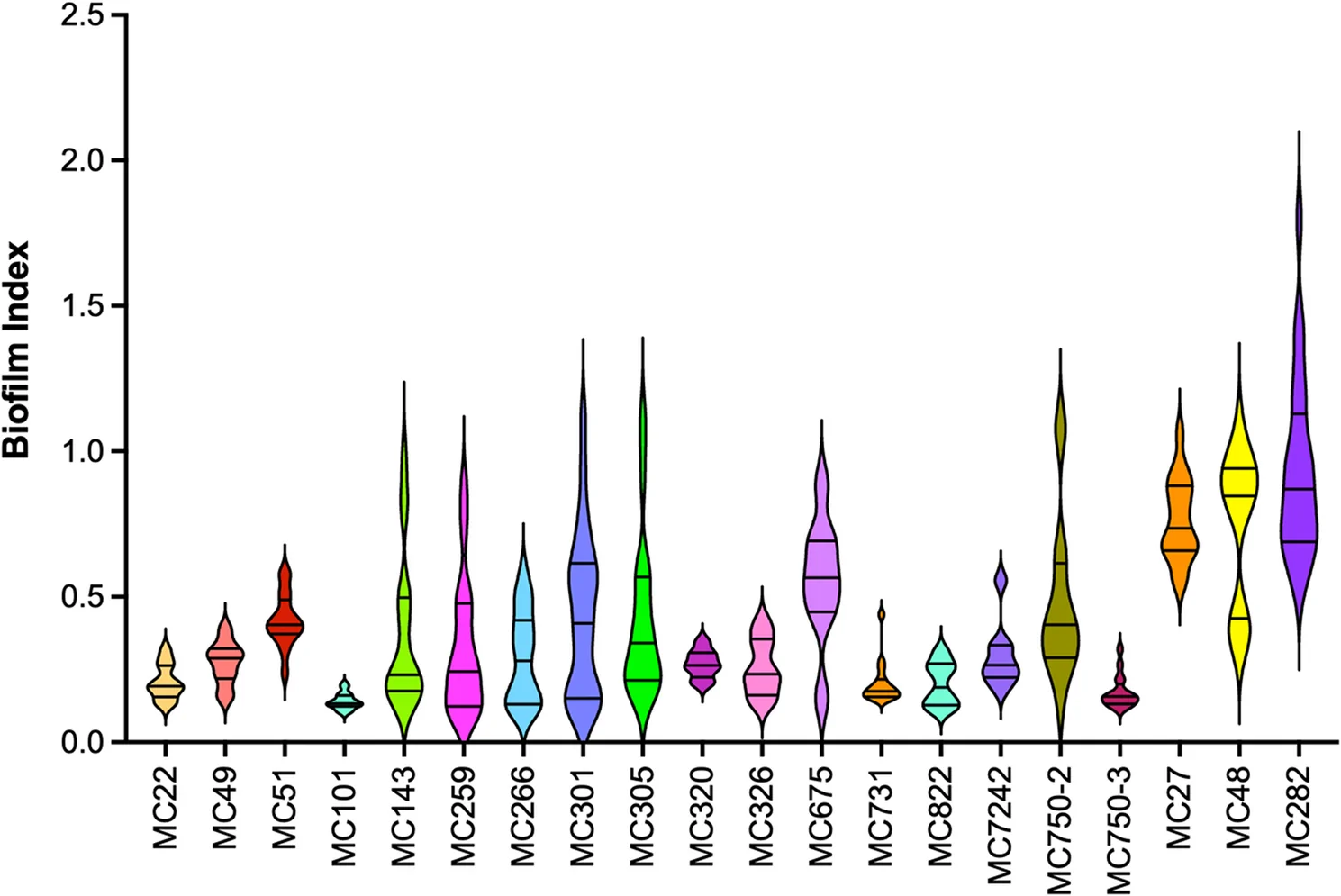
Biofilm-forming capacity of clinical isolates of *M. catarrhalis*. Quantification of biofilm biomass formed by 20 clinical isolates of *M. catarrhalis* after 24 h of incubation, using crystal violet staining in a 96-pin replicator assay. Data represent mean ± SD from at least two independent experiments

After 24 h, *M. catarrhalis* strains exhibited stable biofilm formations, with some isolates showing strong biofilm production. The MC282 strain showed high biofilm forming ability, and it was used for further experiments (Fig. 1).

### Synergistic effect of biofilm formation in co-cultures of NTHi and *M. catarrhalis*

**Fig. 2:**
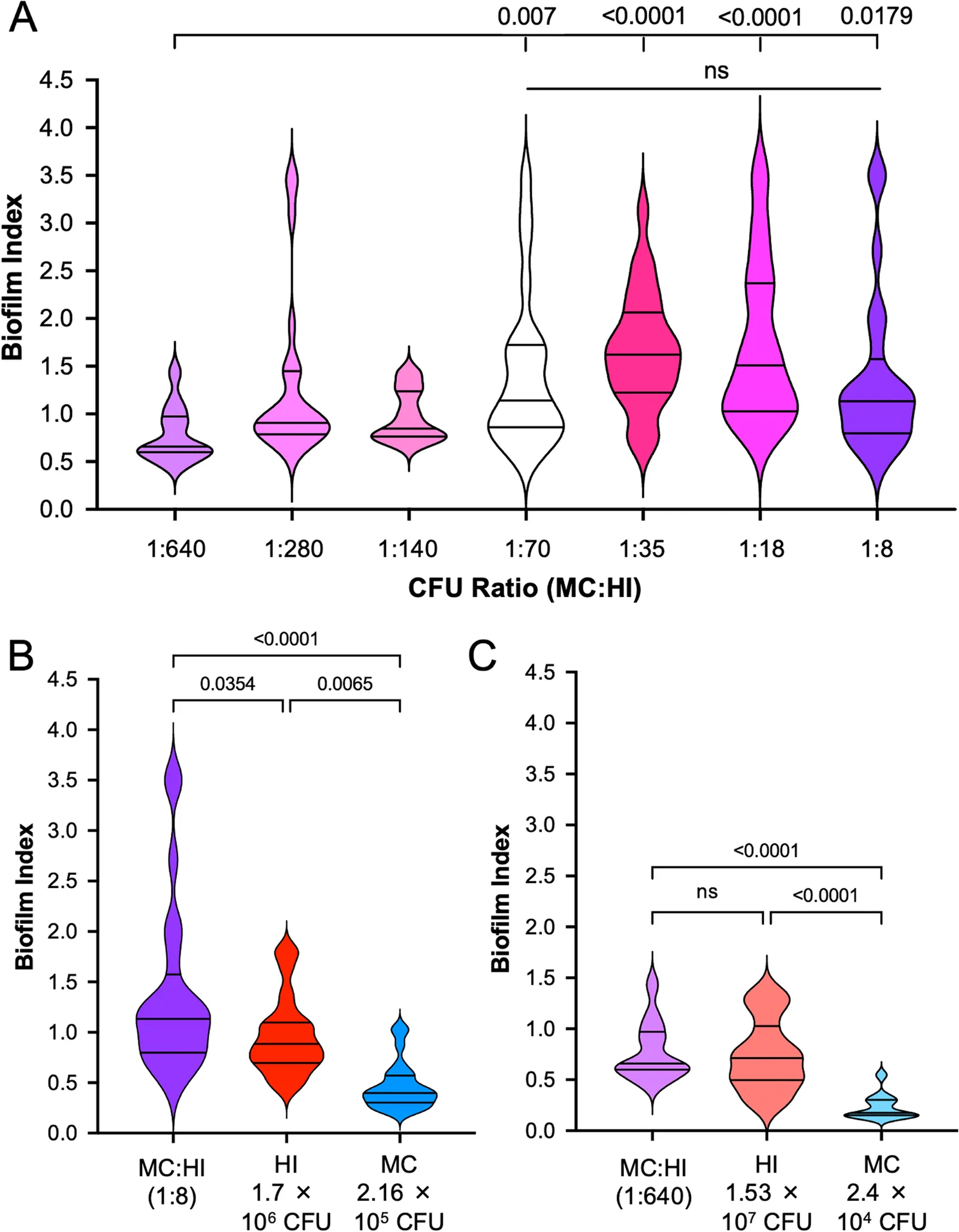
Enhancement of biofilm formation in co-cultures of NTHi and *M. catarrhalis*. **A** Biofilm formation after 24 h of co-culture with varying CFU ratios of *M. catarrhalis* (MC282) to NTHi (IH-202) (ranging from 1:640 to 1:8). **B** Comparison of biofilm production between mono-cultures and co-culture containing approximately 2.16 × 10⁵ CFU of *M. catarrhalis* and 1.7 × 10⁶ CFU of NTHi (approximately 1:8 CFU ratio). Mono-culture controls contained the corresponding CFU numbers of each species alone. **C** Comparison of biofilm production between mono-cultures and co-culture containing approximately 2.4 × 10⁴ CFU of *M. catarrhalis* and 1.53 × 10⁷ CFU of NTHi (approximately 1:640 CFU ratio). Mono-culture controls contained the corresponding CFU numbers of each species alone. Data represent mean ± SD. ns: not significant

**Fig. 3:**
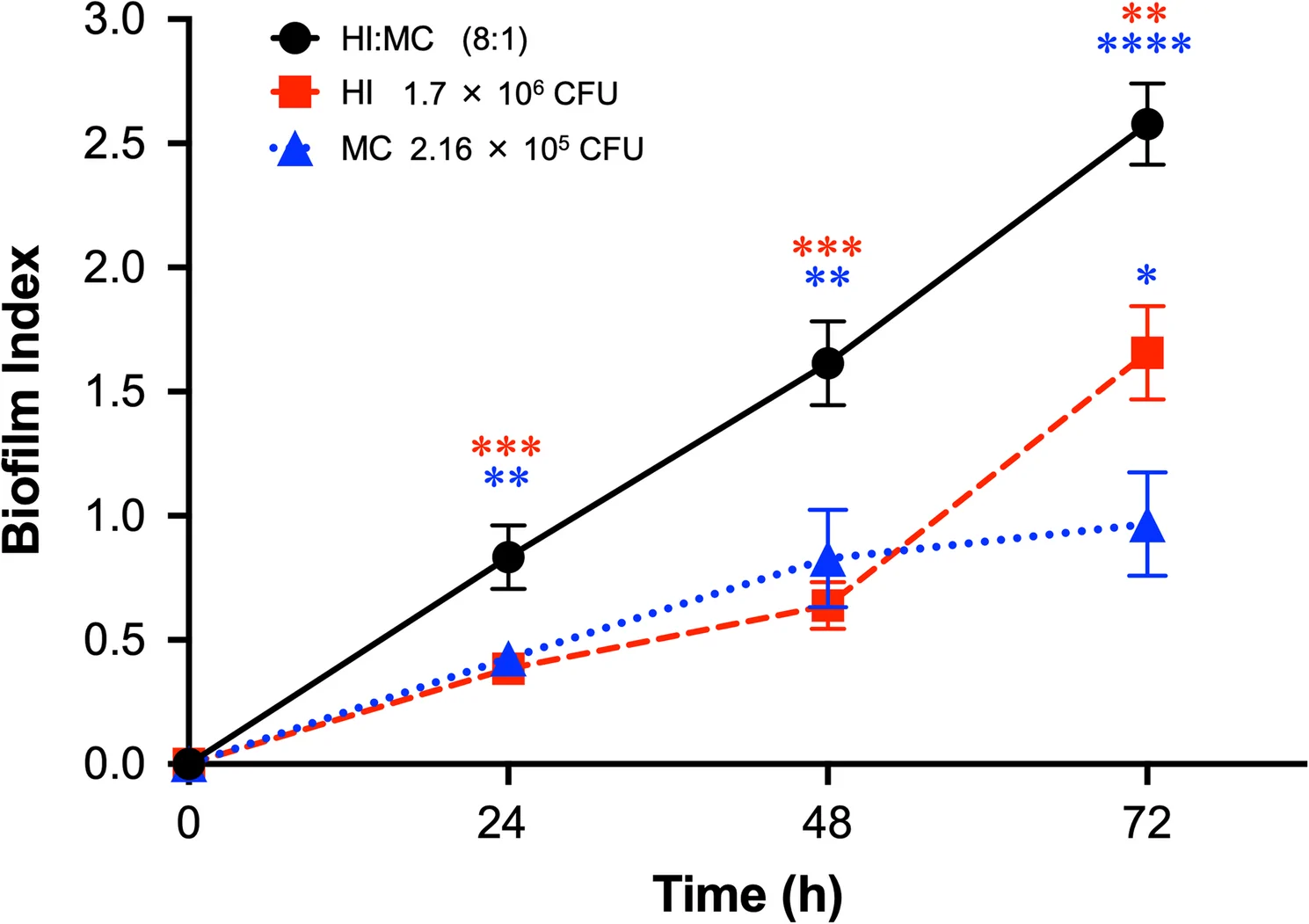
Time-dependent biofilm formation in co-cultures of NTHi and *M. catarrhalis*. Biofilm production measured at 24, 48, and 72 h in mono-cultures of NTHi (IH202), *M. catarrhalis* (MC282), and their co-culture containing approximately 2.16 × 10⁵ CFU of *M. catarrhalis* and 1.7 × 10⁶ CFU of NTHi (approximately 1:8 CFU ratio). Mono-culture controls contained the corresponding CFU numbers of each species alone. Co-culture consistently resulted in significantly greater biofilm formation compared with each species alone. Data represent mean ± SD from three independent experiments. *, *p* < 0.05; **, *p* < 0.01; ***, *p* < 0.001; ****, *p* < 0.0001

Co-culture of MC282 and IH202 showed that increasing the proportion of MC282 (from *M. catarrhalis* to NTHi CFU ratios of 1:640 to 1:8) led to greater enhancement of biofilm formation compared with monocultures (Fig. 2A). Biofilm formation in co-cultures at a *M. catarrhalis* to NTHi CFU ratio of 1:8 was greater than that observed in either MC282 or IH202 monocultures. In contrast, no enhancement of biofilm formation was observed at a *M. catarrhalis* to NTHi CFU ratio of 1:640 (Fig. 2B and C). Although the 1:35 and 1:18 CFU ratios of *M. catarrhalis* to NTHi also showed high biofilm formation, no statistically significant differences were observed compared with the 1:8 condition. Therefore, the condition containing approximately 2.16 × 10^5^ CFU of *M. catarrhalis* and 1.7 × 10^6^ CFU of NTHi (1:8 CFU ratio) was selected for subsequent experiments as a representative high-biofilm-producing condition. Co-cultures of MC282 and IH202 at a *M. catarrhalis* to NTHi CFU ratio of 1:8 produced greater biofilm amounts at 24, 48, and 72 h than monocultures at the same total bacterial load (Fig. 3).

### The effects of antibiotics against biofilms in co-culture of NTHi and *M. catarrhalis*

**Fig. 4:**
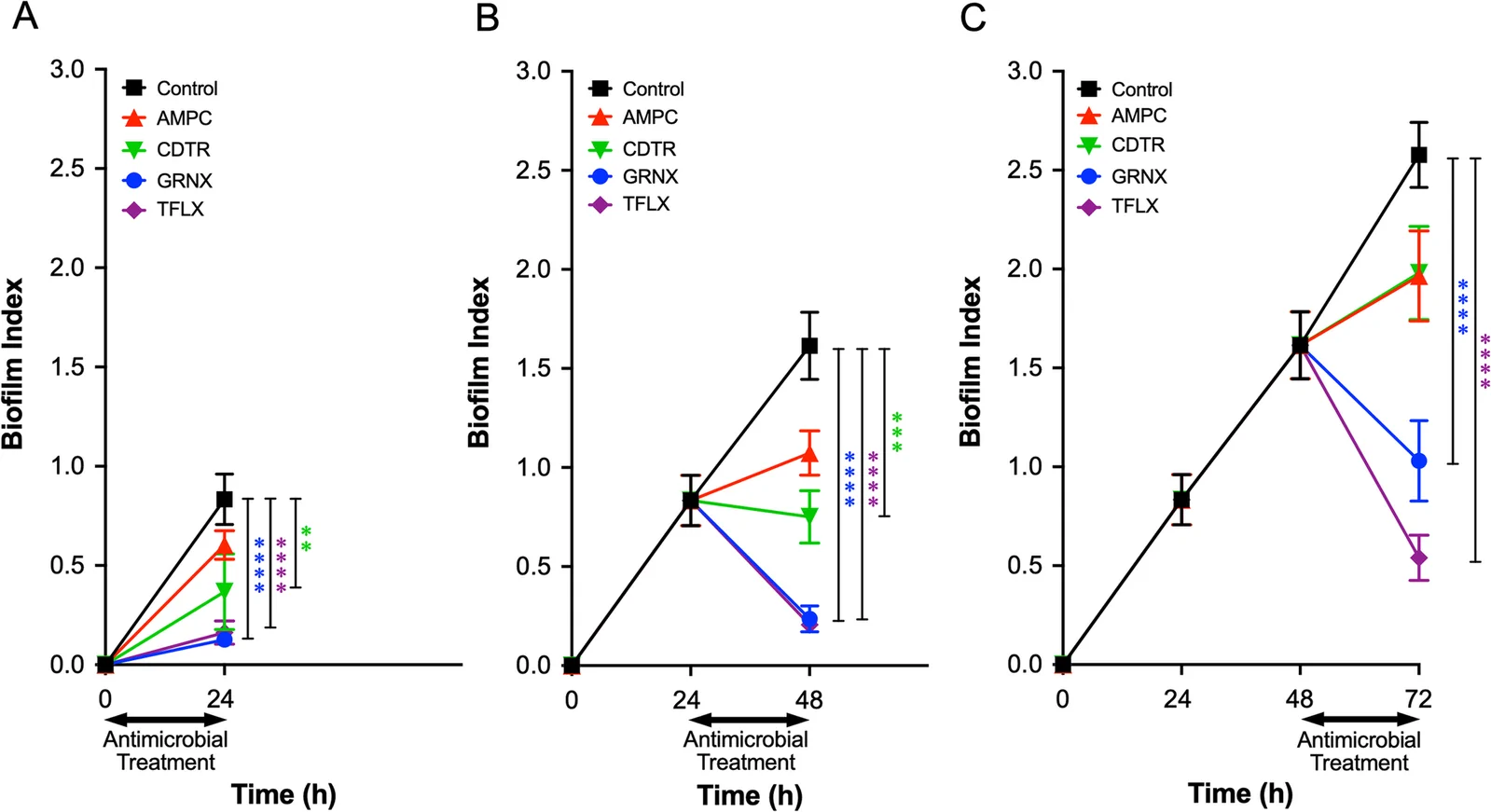
Effect of antibiotics on developing and matured biofilms in co-cultures. **A** Impact of amoxicillin (AMPC), cefditoren pivoxil (CDTR), garenoxacin (GRNX), and tosufloxacin (TFLX) at 1 × MIC on biofilm formation during initial 0–24 h exposure. **B** Effect of the same antibiotics on 24 h-matured biofilms during 24–48 h exposure. **C** Effect of the same antibiotics on 48 h-matured biofilms during 48–72 h exposure. Biofilm production measured at 24, 48, and 72 h in co-culture of NTHi (IH202) and *M. catarrhalis* (MC282) (CFU ratio = 8:1). Antibiotic concentrations correspond to 1 × MIC values for NTHi of each agent: AMPC 0.124 µg/mL; CDTR ≤ 0.0315 µg/mL; GRNX ≤ 0.0315 µg/mL; TFLX 0.0625 µg/mL. **, *p* < 0.01; ***, *p* < 0.001; ****, *p* < 0.0001

At a concentration of 1 × MIC, amoxicillin failed to inhibit biofilm formation in co-cultures during the initial exposure period of 0–24 h. Meanwhile, garenoxacin, tosufloxacin and cefditoren significantly inhibited biofilm formation during this interval for 0–24 h (Fig. 4A). In the 24 h-matured biofilms in co-cultures (24–48 h), amoxicillin only exhibited a biostatic effect, suppressing growth but not reducing biofilm amounts. In co-cultures, both garenoxacin and tosufloxacin successfully suppressed and destroyed the 24 h-matured biofilm (Fig. 4B). Cefditoren showed a modest reduction of 24 h-matured biofilm in co-cultures. For 48 h-matured biofilms in co-cultures (48–72 h), amoxicillin was unable to disrupt biofilms, but garenoxacin and tosufloxacin successfully reduced biofilms (Fig. 3C). At concentrations of 1/2 × MICs, 2 × MICs and the clinical concentrations, the effects of the antibiotics on biofilm formation in co-cultures after initial exposure for 0–72 h were similar to those observed after exposure at 1 × MICs. Both garenoxacin and tosufloxacin significantly decreased the biofilm formation in co-cultures of IH202 and MC282 (Fig.S2).

### Antimicrobial effect against matured biofilms in co-cultures of NTHi and *M. catarrhalis* after amoxicillin exposure

**Fig. 5:**
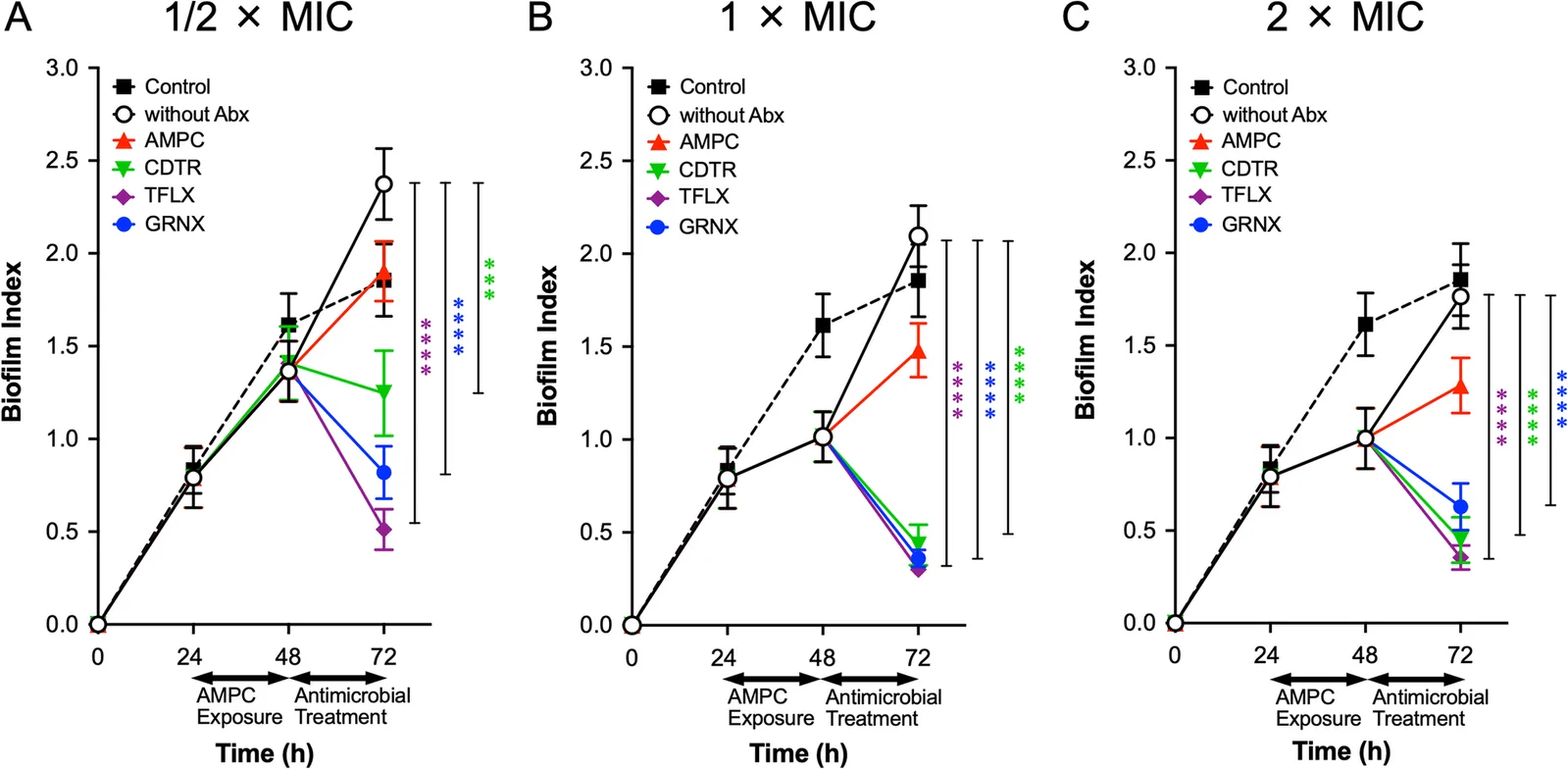
Eradication of amoxicillin-induced matured biofilms in co-cultures by antibiotics. Biofilm after amoxicillin (AMPC) pre-exposure (24–48 h), followed by 24 h exposure (48–72 h) to AMPC, cefditoren pivoxil (CDTR), garenoxacin (GRNX), and tosufloxacin (TFLX) at 1/2 × MIC (**A**), 1 × MIC (**B**), or 2 × MIC (**C**). “Control” indicates biofilms that received neither AMPC pre-exposure during 24–48 h nor antibiotic treatment during 48–72 h. “Without Abx” indicates biofilms that were pre-exposed to AMPC during 24–48 h but received no antibiotic treatment during 48–72 h. Biofilm production measured at 24, 48, and 72 h in co-culture of NTHi (IH202) and *M. catarrhalis* (MC282) (CFU ratio = 8:1). Antibiotic concentrations correspond to 1 × MIC values for NTHi of each agent: AMPC 0.124 µg/mL; CDTR ≦ 0.0315 µg/mL; GRNX ≦ 0.0315 µg/mL; TFLX 0.0625 µg/mL. Data represent mean ± SD. ***, *p* < 0.001; ****, *p* < 0.0001

We further evaluated the effects of antibiotics on mature biofilms in co-cultures of IH202 and MC282 with prior exposure to various concentrations of amoxicillin for 24 h (24–48 h). When the matured biofilm exposed to amoxicillin in co-cultures of IH202 and MC282 was subsequently exposed to antibiotics at 1/2 × MIC concentrations for 24 h (48–72 h), amoxicillin could not reduce biofilms. In contrast, garenoxacin, tosufloxacin and cefditoren could significantly reduce biofilms (Fig. 5A). When the amoxicillin-pretreated biofilms were exposed at 1 × MIC and 2 × MIC concentrations, garenoxacin, tosufloxacin and cefditoren significantly reduced biofilms but amoxicillin did not (Fig. 5B and C). Although exposure to amoxicillin at the adult’s sinus concentration for 24–48 h could reduce the mature biofilms in co-cultures of IH202 and MC282, both suspension (at 48–72 h) or continuous (i.e., 24–72 h) amoxicillin exposure led to further accumulations of biofilm. The effect of amoxicillin pretreatment (i.e., exposure at 24–48 h) on the biofilm was rendered ineffective. However, the amoxicillin-induced mature biofilms in co-cultures were reduced and disrupted by exposure to garenoxacin for the 48–72 h after earlier amoxicillin exposure (Fig.S3A). In contrast, the mature biofilm was reduced by continuous exposure to amoxicillin at the child’s middle ear concentration. Exposure to tosufloxacin for 48–72 h after prior amoxicillin exposure also reduced and disrupted the mature biofilm (Fig.S3B).

### Survival of NTHi and *M. catarrhalis* in biofilms in co-cultures after antimicrobial exposures

**Fig. 6:**
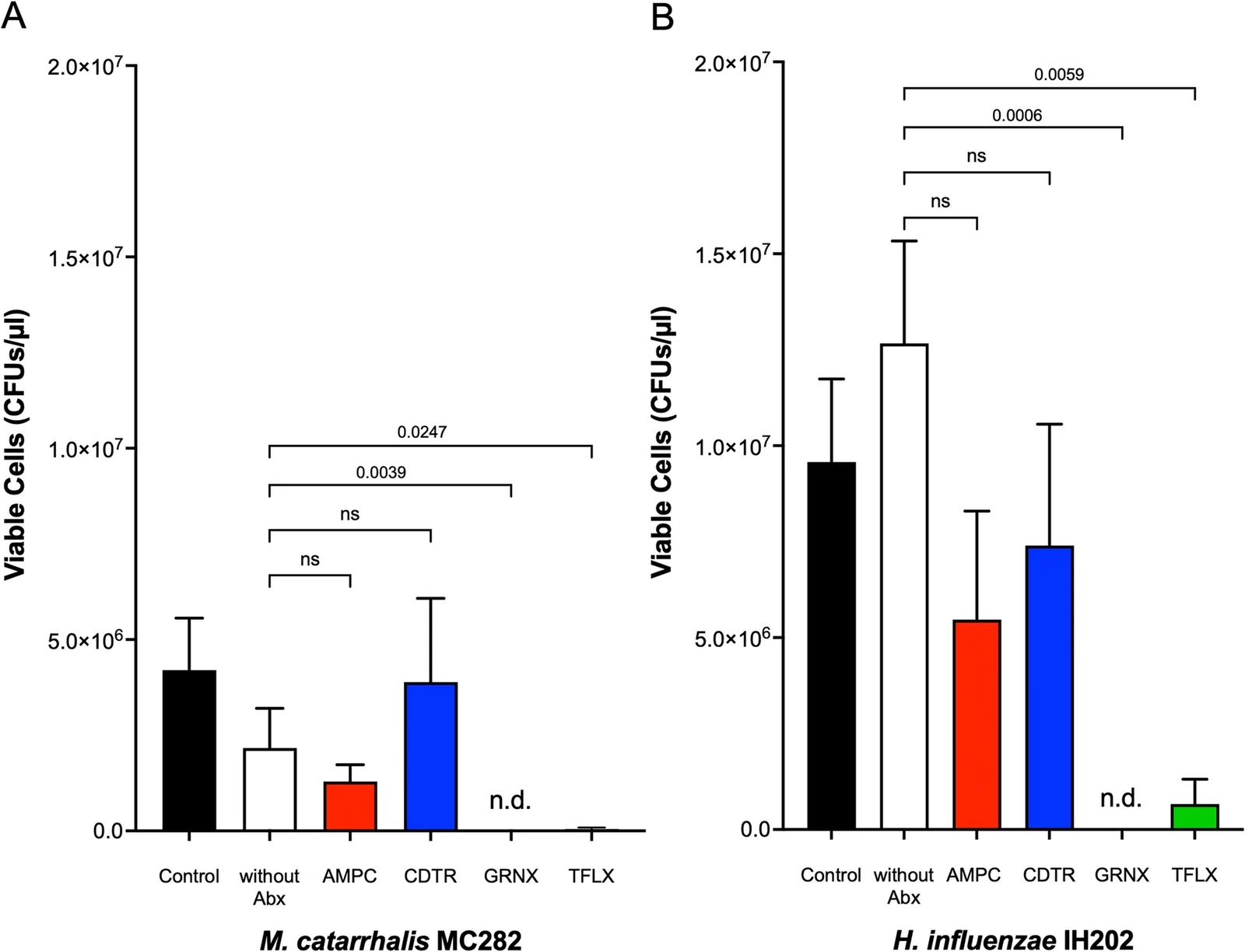
Viability of NTHi and *M. catarrhalis* in 24 h-matured biofilms in co-cultures following antimicrobial treatment. Viable cells of *M. catarrhalis* (**A**) and NTHi (**B**) recovered from 24 h-matured biofilms in co-cultures after 24 h treatment with amoxicillin (AMPC), cefditoren pivoxil (CDTR), garenoxacin (GRNX), and tosufloxacin (TFLX) at 1 × MIC concentrations were counted, respectively. “Control” indicates biofilms that received neither AMPC pre-exposure during 24–48 h nor antibiotic treatment during 48–72 h. “Without Abx” indicates biofilms that were pre-exposed to AMPC during 24–48 h but received no antibiotic treatment during 48–72 h. Biofilm production measured at 24, 48, and 72 h in co-culture of NTHi (IH202) and *M. catarrhalis* (MC282) (CFU ratio = 8:1). Antibiotic concentrations correspond to 1 × MIC values for NTHi of each agent: AMPC 0.124 µg/mL; CDTR ≤ 0.0315 µg/mL; GRNX ≤ 0.0315 µg/mL; TFLX 0.0625 µg/mL. Bars represent mean ± SD. n.d.: not detected

Under exposure to antibiotics at 1 × MIC concentrations, garenoxacin and tosufloxacin could kill IH202 and MC282 in 24 h-matured biofilms, but amoxicillin and cefditoren could not (Fig. 6). No viable bacteria were recovered after 48–72 h exposure to garenoxacin at 1 × MIC.

## Discussion

Increased biofilm formation by pathogenic bacteria is recognized as a risk factor for persistent or recurrent AOM following antimicrobial treatment. A hallmark of bacteria grown in biofilms is their increased resistance to antibiotics [29, 35]. In this study, dual species demonstrated significantly more biofilms and resistance to amoxicillin than monocultures, particularly when *M. catarrhalis* was present in high proportion. In polymicrobial biofilms, such structural organization is considered to provide a protective niche that limits antibiotic penetration and enhances survival against antimicrobial exposure, thereby contributing directly to antibiotic tolerance.

Multispecies biofilms are known to form stable microconsortia, to promote the establishment of physicochemical gradients, to facilitate horizontal gene transfer and to enhance cell-to-cell communication [36]. Biofilm formation in co-culture models with *Streptococcus pneumoniae* and *H. influenzae* reportedly varies depending on the strain combinations used [37, 38]. Consistent with prior findings, we found that NTHi forms more robust biofilm in co-cultures with *M. catarrhalis* than in monocultures [39]. The underlying mechanisms require further elucidation, however, several potential mechanisms may explain the enhanced biofilm formation observed in co-culture. In addition to β-lactamase-mediated protection via outer membrane vesicles, *M. catarrhalis* may contribute to biofilm maturation by providing structural support within the extracellular matrix or by stabilizing biofilm architecture, thereby indirectly enhancing NTHi persistence. Previous studies have shown that AI-2–mediated signaling from NTHi promotes *M. catarrhalis* biofilm development [40, 41]. Although the precise mechanisms underlying the enhanced biofilm formation observed in the present study remain unclear, these findings suggest that interspecies interactions may contribute to the stability and antimicrobial tolerance of dual-species biofilms. These findings suggest that both biochemical interactions and physical associations may contribute to the enhanced stability and antimicrobial tolerance observed in dual-species biofilms. In addition, structural analyses have shown that NTHi often surrounds dense *M. catarrhalis* aggregates, facilitating cooperative persistence within polymicrobial biofilms [37–40].

Next, we observed reduced efficacy of amoxicillin against biofilms formed by co-cultures of NTHi and *M. catarrhalis*. The sequential exposure design was intended to approximate a stepwise antimicrobial response scenario in which bacteria initially encounter amoxicillin and are subsequently exposed to the same or alternative agents after incomplete eradication. This approach allows assessment of how prior β-lactam exposure influences the susceptibility of established polymicrobial biofilms under experimental conditions [42]. In the present study, the MC282 strain was confirmed to produce β-lactamase using a nitrocefin-based assay. This finding suggests that enzymatic degradation may contribute to the reduced activity of amoxicillin within the polymicrobial biofilm environment. However, we did not include a β-lactamase–negative *M. catarrhalis* strain or perform quantitative analyses of β-lactamase activity, therefore, the relative contribution of enzymatic degradation versus biofilm-associated tolerance cannot be determined. Therefore, the reduced efficacy of amoxicillin observed in this model cannot be explained solely by the relative abundance of β-lactamase–producing *M. catarrhalis*, but likely reflects combined effects of enzymatic activity and biofilm-associated tolerance mechanisms. These findings suggest that even a relatively small proportion of β-lactamase–producing bacteria may influence the overall antimicrobial susceptibility within polymicrobial biofilms.

Previous studies have shown that β-lactamase-containing OMVs released from *M. catarrhalis* can protect neighboring bacteria by inactivating β-lactam antibiotics [43]. In addition to acting as an indirect pathogen through β-lactamase production, *M. catarrhalis* has been reported to be associated with enhanced NTHi persistence and biofilm formation in polymicrobial settings [37, 40]. Interspecies bacterial communication contributes to persistence and antimicrobial resistance and is considered important in the pathogenesis of AOM [40]. We previously demonstrated that amoxicillin at both sub-MIC and MIC levels promoted the biofilm formation of NTHi, but that it was less effective at eradicating them. Garenoxacin and tosufloxacin have been shown to be effective in eradicating mature amoxicillin-induced NTHi biofilms and eliminating viable NTHi within them [11, 44]. Similarly, others reported that sub-inhibitory concentrations of β-lactams stimulate NTHi biofilm formation, which further highlights the limitations of amoxicillin [11, 42]. Another study showed that levofloxacin significantly reduced NTHi biofilm, whereas amoxicillin did not [25]. Tosufloxacin has been shown to be effective against NTHi biofilms, and it has also demonstrated bactericidal activity against intracellular NTHi in a human epithelial model [31, 44]. Taken together, these findings indicate reduced activity of amoxicillin in this experimental biofilm model and suggest differential antimicrobial responses depending on drug class. Therefore, the reduced efficacy of amoxicillin observed in the co-culture biofilms may reflect a combination of enzymatic degradation by β-lactamase produced by *M. catarrhalis* and intrinsic biofilm-associated tolerance, rather than biofilm structure alone.

Although the NTHi strain used in this study (IH202) is a β-lactamase–nonproducing isolate, the prevalence of β-lactamase producing NTHi varies considerably by geographic region. In many countries, including North America, Europe, and the Middle East, β-lactamase producing strains constitute a substantial proportion of clinical isolates, whereas in Japan most NTHi isolates remain β-lactamase-nonproducing, with resistance more commonly mediated by altered penicillin-binding proteins [45–48]. Therefore, in many clinical settings amoxicillin is administered in combination with clavulanate to inhibit enzymatic degradation. Nevertheless, treatment failure is still observed in infections involving polymicrobial biofilms. Our findings suggest that reduced susceptibility in the present model cannot be explained solely by enzymatic inactivation, but also reflects biofilm-associated tolerance mechanisms such as restricted antibiotic penetration, metabolic heterogeneity, and the presence of persister cells. Accordingly, even in situations where β-lactamase activity is pharmacologically inhibited, coexistence with *M. catarrhalis* may promote NTHi persistence through biofilm-mediated protection. These observations support the concept that polymicrobial biofilm formation itself represents an independent barrier to eradication beyond classical β-lactamase-mediated resistance.

Finally, we demonstrated a reduction in the antimicrobial efficacy against NTHi and *M. catarrhalis* co-existing within biofilms. *M. catarrhalis* was previously shown to enhance the survival of NTHi, even in the presence of *S. pneumoniae* in triple species biofilms, which is commonly found in the human nasopharynx. *M. catarrhalis* contributes to the formation of a stable polymicrobial biofilm by promoting NTHi survival and by facilitating co-colonization with other otopathogens [37]. Bacteria embedded in biofilms often persist despite antibiotic exposure. In particular, NTHi within mature biofilms was not eradicated by amoxicillin unless exposed to high concentrations, which supports previous suggestions that this resistance may be attributed to the presence of persistent cells [44]. In contrast, quinolones, which can penetrate the exopolysaccharide matrix, reportedly exhibited bactericidal activity against biofilm-associated bacteria across various growth phases, including the sessile state [25, 44, 49–52]. Although quinolone exposure resulted in a marked reduction of biofilm biomass, we did not investigate AI-2 production or other interspecies signaling pathways in the present study. Therefore, we cannot exclude the possibility that intrinsic dispersal signaling contributed to the observed reduction in biofilm biomass. Accordingly, the decrease observed after quinolone exposure should not be interpreted as evidence of a direct biofilm-disrupting effect of these antibiotics alone. Rather, it may reflect a combination of bactericidal activity and endogenous dispersal mechanisms, including AI-2-mediated signaling or OMVs associated β-lactamase-related interactions reported in previous studies [40, 41]. Further mechanistic analyses are required to delineate these potential contributions.

These findings suggest that polymicrobial conditions may influence antimicrobial responsiveness compared with single-species models. Recent studies have further emphasized that interspecies interactions within respiratory polymicrobial biofilms can modulate bacterial survival strategies, virulence expression, and antimicrobial tolerance, thereby contributing to persistent infection in the upper airway. Such observations support the clinical relevance of evaluating antibiotic efficacy under polymicrobial conditions rather than relying solely on single-species biofilm models [53].

Treatment guidelines for upper respiratory tract infections including AOM and acute rhinosinusitis typically recommend amoxicillin as the first-line antimicrobial agent. However, exposure to subinhibitory concentrations of amoxicillin has been shown to promote biofilm formation by various microorganisms. Integrated transcriptomic and metabolomic analyses have identified amino sugar and nucleotide sugar metabolism as key regulatory pathways involved in this process [54]. In contrast, respiratory quinolones such as garenoxacin and tosufloxacin were associated with a greater reduction in viable NTHi within biofilms under the experimental conditions used in this study. These observations warrant further investigation in more clinically relevant models of biofilm-associated NTHi infection.

This study has several limitations. We primarily quantified biofilm formation and did not perform detailed structural or microscopic analyses of the biofilms, therefore, the underlying mechanisms and spatial organization of polymicrobial biofilms were not fully evaluated. Although microscopic characterization of NTHi biofilms has been reported in previous studies [9, 11, 55], we did not perform microscopic analyses of biofilms under either static or shaking conditions in the present study. In addition, biofilms were generated under shaking conditions to ensure reproducible biofilm formation. However, this does not fully replicate the in vivo microenvironment, and biofilm architecture may differ under static conditions. Second, the efficiency of biofilm detachment was not quantitatively assessed after agitation, and pins were not examined microscopically following recovery. However, this procedure was applied consistently across all experimental conditions to allow relative comparisons. Furthermore, although experiments were repeated independently at least twice with consistent findings, additional biological replicates would further strengthen the robustness of the observations and allow a more comprehensive assessment of biological variability. In addition, our experimental model evaluated the activity of amoxicillin alone and did not include a β-lactam/β-lactamase inhibitor combination such as amoxicillin-clavulanate, which is commonly used in clinical practice. Therefore, the present findings may not fully reflect antimicrobial responses under clinical treatment conditions.

Co-culture of NTHi and *M. catarrhalis* was associated with significantly enhanced biofilm formation as the proportion of *M. catarrhalis* increased. This augmented biofilm formation was accompanied by reduced susceptibility to amoxicillin under the tested conditions. Our findings suggest that *M. catarrhalis* may contribute not only as a pathogen of upper respiratory tract infections but also as a facilitator of NTHi persistence within polymicrobial biofilms, potentially limiting antibiotic efficacy and host clearance. Such interactions may contribute to persistence under experimental conditions. Respiratory quinolones such as garenoxacin and tosufloxacin were associated with greater reductions in biofilm biomass and viable bacteria in this experimental model, warranting further investigation in more clinically relevant settings. To our knowledge, few studies have systematically evaluated sequential β-lactam exposure followed by alternative antimicrobial treatment within a controlled dual-species biofilm framework.

## Conclusions

This study demonstrated that co-culture of NTHi and *M. catarrhalis* significantly enhanced biofilm formation, particularly when *M. catarrhalis* was present in higher proportions, resulting in markedly reduced susceptibility to amoxicillin. These findings suggest that *M. catarrhalis* not only acts as a pathogen in upper respiratory tract infections but also facilitates NTHi persistence within polymicrobial biofilms by promoting biofilm maturation and antimicrobial tolerance. The diminished efficacy of amoxicillin under these conditions highlights a limitation of conventional first-line therapies for acute otitis media and related infections. In contrast, respiratory quinolones produced larger reductions in biofilm biomass and viable bacteria in the co-culture model, supporting the notion that polymicrobial biofilm context influences antimicrobial responsiveness.

## Supplementary Information

Supplementary Material 1: Supplemental Fig. 1. Colony morphology of *M. catarrhalis* and *H. influenzae*. Representative colony morphologies used to differentiate the two species during enumeration from mixed-species biofilms. After incubation on chocolate agar plates for 18–20 h at 37 °C under 5% CO₂, *M. catarrhalis* formed opaque, white colonies (A), whereas NTHi formed smaller, greyish, and translucent colonies (B).

Supplementary Material 2: Supplemental Fig. 2. Antibiotic effects on biofilms in co-cultures at various MICs and clinical concentrations. Evaluation of amoxicillin (AMPC), cefditoren pivoxil (CDTR), garenoxacin (GRNX), and tosufloxacin (TFLX) on NTHi–*M. catarrhalis* biofilm formation across 1/2 × MIC (A), 2 × MIC (B), and clinical concentrations (C). AMPC (Low) and AMPC (High) indicate adult’s sinus concentration (0.2 µg/ml) and child’s middle ear fluid (MEF) concentration (5.8 µg/ml), respectively. Biofilm production measured at 24, 48, and 72 h in co-culture of NTHi (IH202) and *M. catarrhalis* (MC282) (CFU ratio = 8:1). *, *p* < 0.05; **, *p* < 0.01; ***, *p *< 0.001; ****, *p* < 0.0001.

Supplementary Material 3: Supplemental Fig. 3. Clinical concentration effect of antibiotics on AMPC-pretreated biofilms. Evaluation of biofilm formation after 24–48 h amoxicillin (AMPC) exposure, followed by 48–72 h treatment with AMPC, cefditoren pivoxil (CDTR), garenoxacin (GRNX), or tosufloxacin (TFLX) at clinical concentrations. “Control” indicates biofilms that received neither AMPC pre-exposure during 24–48 h nor antibiotic treatment during 48–72 h. “Without Abx” indicates biofilms that were pre-exposed to AMPC during 24–48 h but received no antibiotic treatment during 48–72 h. Biofilm production measured at 24, 48, and 72 h in co-culture of NTHi (IH202) and *M. catarrhalis* (MC282) (CFU ratio = 8:1). (A) Adult’s sinus concentrations. (B) Child’s middle ear concentrations. *, *p* < 0.05; ****, *p* < 0.0001.

## Data Availability

All data generated or analyzed during this study are included in this published article and its supplementary information files.
